# A223 THE CHAIR STAND TEST IS A RELIABLE FRAILTY METRIC FOR PREDICTING WAITLIST MORBIDITY AND MORTALITY IN PATIENTS WITH CIRRHOSIS

**DOI:** 10.1093/jcag/gwab049.222

**Published:** 2022-02-21

**Authors:** V V Nguyen, S Wang, R Whitlock, C Xu, S Taneja, S Singh, J Abraldes, K Burak, R Bailey, J Lai, P Tandon

**Affiliations:** 1 Faculty of Medicine, University of Alberta, Edmonton, AB, Canada; 2 Gastroenterology, University of Alberta, Edmonton, SK, Canada; 3 University of Alberta, Edmonton, AB, Canada; 4 Royal Alexandra Hospital, Edmonton, AB, Canada; 5 Chronic Disease Intervention Centre, Winnipeg, MB, Canada; 6 Department of Medicine, San Francisco, CA; 7 Department of Hepatology, Chandigarh, India; 8 Liver Unit, Division of Gastroenterology and Hepatology, Calgary, AB, Canada; 9 Division of Gastroenterology and Hepatology, San Francisco, CA

## Abstract

**Background:**

Frailty is defined as a clinical state of increased vulnerability to health and age associated stressors. The liver frailty index (LFI), composed of grip strength, chair stand and balance testing, is an accepted predictor of morbidity and mortality in cirrhosis. With the need for COVID-19 related social distancing, many appointments are being carried out virtually. The chair stand subcomponent of the LFI has the potential to be evaluated virtually, with a high reliability as compared to in-person testing noted in other disease populations.

**Aims:**

To determine if the chair stand test is an independent predictor of morbidity and mortality in patients with cirrhosis.

**Methods:**

822 adult patients with cirrhosis were prospectively enrolled from five centers (3 in Canada, 1 in the United States, and 1 in India). Inclusion criteria included adult patients with cirrhosis. 787 of these patients completed a chair stand test at baseline, measured as the time (seconds) a patient takes to rise from sitting with their arms folded across their chest five times (measured in-person). The times were divided into 3 categories: >15 seconds, between 10 and 15 seconds, and <10 seconds. Patients who could not complete 5 chair stands were classified in the >15 seconds category. Primary outcome was all-cause mortality. Secondary outcome was unplanned all-cause hospital admission. Fine-Gray proportional hazard regression models were used to evaluate the association between the chair stand time and the outcomes. We adjusted for baseline age, sex, and MELD score and accounted for liver transplantation as a competing risk. Cumulative incidence functions were used to create a graphical representation of the survival analysis.

**Results:**

Patients were divided into three groups: group 1, <10 seconds (n = 276); group 2, 10–15 seconds (n = 290); and group 3, >15 seconds (n = 221). Mortality was increased in group 3 in comparison to group 1 (HR 3.21, 95% CI: 2.16–4.78, p<0.001). Similarly, the hazard of non-elective hospitalizations was higher in group 3 in comparison to group 1 (HR 2.24, 95% CI: 1.73–2.91, p<0.001). Overall, patients with chair stand times greater than 15 seconds had increased all-cause mortality (HR 2.78, 95% CI 2.01–3.83, p<0.001) and non-elective hospitalizations (HR 1.84, 95% CI 1.48–2.29, p<0.001) when compared to patients with times less than 15 seconds.

**Conclusions:**

A time to complete 5 chair stands of >15 seconds predicts morbidity and mortality in patients with cirrhosis. This test shows promise as a frailty measure that could be evaluated over a virtual platform.

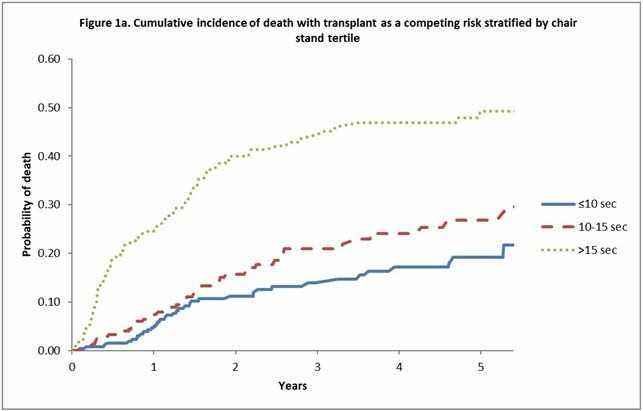

**Funding Agencies:**

None

